# Sex differences among subcutaneous implantable cardioverter-defibrillator recipients: a propensity-matched, multicentre, international analysis from the i-SUSI project

**DOI:** 10.1093/europace/euae115

**Published:** 2024-05-02

**Authors:** Marco Schiavone, Alessio Gasperetti, Julia Vogler, Paolo Compagnucci, Mikael Laredo, Alexander Breitenstein, Simone Gulletta, Martin Martinek, Lukas Kaiser, Fabrizio Tundo, Pietro Palmisano, Giovanni Rovaris, Antonio Curnis, Jürgen Kuschyk, Mauro Biffi, Roland Tilz, Luigi Di Biase, Claudio Tondo, Giovanni B Forleo, i-SUSI investigators, A Gasperetti, R Arosio, M Viecca, G B Forleo, M Schiavone, F Tundo, M Moltrasio, C Tondo, M Ziacchi, I Diemberger, A Angeletti, M Biffi, N Fierro, S Gulletta, P Della Bella, G Mitacchione, A Curnis, P Compagnucci, M Casella, A Dello Russo, L Santini, C Pignalberi, M Magnocavallo, A Piro, C Lavalle, F Picarelli, D Ricciardi, E Bressi, L Calò, E Montemerlo, G Rovaris, S De Bonis, A Bisignani, G Bisignani, G Russo, E Pisanò, P Palmisano, F Guarracini, F Vitali, M Bertini, J Vogler, T Fink, R Tilz, F Fastenrath, J Kuschyk, L Kaiser, S Hakmi, M Laredo, X Waintraub, E Gandjbakhch, N Badenco, A Breitenstein, A M Saguner, M Martine, S Seidl, X Zhang, L Di Biase

**Affiliations:** Department of Clinical Electrophysiology & Cardiac Pacing, Centro Cardiologico Monzino, IRCCS, Via Carlo Parea 4, 20138 Milan, Italy; Department of Cardiology, Johns Hopkins University, Baltimore, USA; Department of Rhythmology, University Heart Center Lübeck, Lubeck, Germany; Cardiology and Arrhythmology Clinic, University Hospital ‘Ospedali Riuniti’, Ancona, Italy; Institut de Cardiologie, Groupe Hospitalier Pitié-Salpêtrière and Sorbonne Université, Paris, France; Cardiology Clinic, University Hospital Zurich, Zurich, Switzerland; Arrhythmology and Electrophysiology Unit, San Raffaele Hospital, IRCCS, Milan, Italy; Department of Internal Medicine 2/Cardiology, Ordensklinikum Linz Elisabethinen, Linz, Austria; Department of Cardiology and Critical Care Medicine, St. George Klinik Asklepios, Hamburg, Germany; Department of Clinical Electrophysiology & Cardiac Pacing, Centro Cardiologico Monzino, IRCCS, Via Carlo Parea 4, 20138 Milan, Italy; Cardiology Unit, ‘Card. G. Panico’ Hospital, Tricase, Italy; Cardiology Unit, Fondazione IRCCS San Gerardo dei Tintori, Monza, Italy; Cardiology Unit, Spedali Civili Brescia, Brescia, Italy; Cardiology Unit, University Medical Centre Mannheim, Manheim, Germany; Cardiology Unit, IRCCS, Department of Experimental, Diagnostic and Specialty Medicine, Sant’Orsola Hospital, University of Bologna, Bologna, Italy; Department of Rhythmology, University Heart Center Lübeck, Lubeck, Germany; Cardiac Arrhythmia Center, Division of Cardiology at Montefiore-Einstein Center, Bronx, New York, USA; Department of Clinical Electrophysiology & Cardiac Pacing, Centro Cardiologico Monzino, IRCCS, Via Carlo Parea 4, 20138 Milan, Italy; Department of Biomedical, Surgical and Dental Sciences, University of Milan, Milan, Italy; Cardiology Unit, Luigi Sacco University Hospital, Milan, Italy

**Keywords:** S-ICD, Sex differences, Gender differences, Sudden cardiac death, Appropriate shocks

## Abstract

**Aims:**

Women have been historically underrepresented in implantable cardioverter-defibrillator (ICD) trials. No data on sex differences regarding subcutaneous ICDs (S-ICD) carriers have been described. Aim of our study was to investigate sex-related differences among unselected S-ICD recipients.

**Methods and results:**

Consecutive patients enrolled in the multicentre, international i-SUSI registry were analysed. Comparisons between sexes were performed using a 1:1 propensity matching adjusted analysis for age, body mass index (BMI), left ventricular function, and substrate. The primary outcome was the rate of appropriate shocks during follow-up. Inappropriate shocks and other device-related complications were deemed secondary outcomes. A total of 1698 patients were extracted from the i-SUSI registry; 399 (23.5%) were females. After propensity matching, two cohorts of 374 patients presenting similar baseline characteristics were analysed. Despite similar periprocedural characteristics and a matched BMI, women resulted at lower risk of conversion failure as per PRAETORIAN score (73.4% vs. 81.3%, *P* = 0.049). Over a median follow-up time of 26.5 [12.7–42.5] months, appropriate shocks were more common in the male cohort (rate/year 3.4% vs. 1.7%; log-rank *P* = 0.049), while no significant differences in device-related complications (rate/year: 6.3% vs. 5.8%; log-rank *P* = 0.595) and inappropriate shocks (rate/year: 4.3% vs. 3.1%; log-rank *P* = 0.375) were observed. After controlling for confounders, sex remained significantly associated with the primary outcome (aHR 1.648; CI 0.999–2.655, *P* = 0.048), while not resulting predictor of inappropriate shocks and device-related complications.

**Conclusion:**

In a propensity-matched cohort of S-ICD recipients, women are less likely to experience appropriate ICD therapy, while not showing higher risk of device-related complications.

**Clinical trial registration:**

ClinicalTrials.gov Identifier: NCT0473876.

## Graphical Abstract

**Graphical Abstract:**
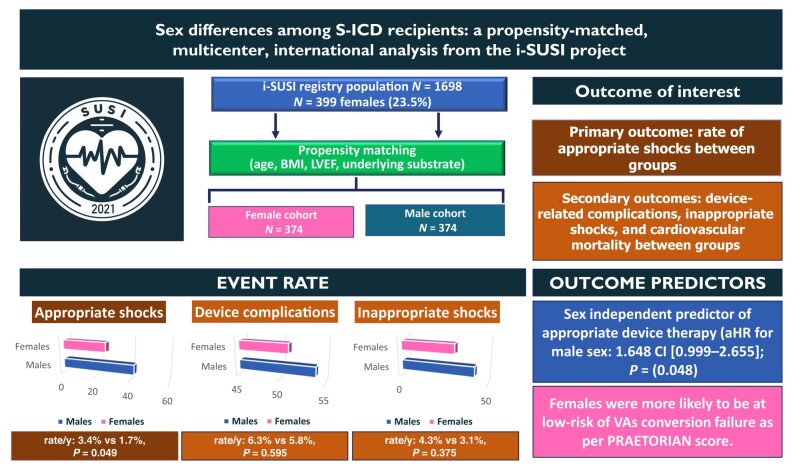

**What’s new?**

- In a large, multicentre, real-world subcutaneous implantable cardioverter-defibrillator (S-ICD) registry, women were significantly underrepresented when compared to their male counterpart.
- Male S-ICD carriers were more likely to experience appropriate device therapy.
- Subcutaneous implantable cardioverter-defibrillator-related complications and inappropriate shocks rate were comparable between sexes.
- Female S-ICD carriers were more likely to be at low-risk of conversion failure as per PRAETORIAN score.

## Introduction

The use of a subcutaneous implantable cardioverter-defibrillator (ICD) is an established therapy for preventing sudden cardiac death in selected patients, serving as an alternative to transvenous (TV) ICDs for individuals who do not require pacing or cardiac resynchronization therapy, regardless of whether they have ischaemic heart failure^1^ or non-ischaemic cardiomyopathies.^2^ Previous research on patients with ICDs has presented conflicting findings regarding the correlation between sex and the occurrence of appropriate device therapy, as well as overall mortality.^3^ While several studies have documented a reduced incidence of ICD therapy among female patients, others have found no disparity in the risk of appropriate ICD therapy between genders.^4,5^ The reasons behind these conflicting findings have been poorly understood, as women have been underrepresented in previous landmark trials regarding ICD therapy, generally accounting for ∼30% of enrolled patients.^6^ However, whether women are underrepresented in clinical trials or truly undertreated compared to their male counterparts due to sex differences in the epidemiology and presentation of ventricular arrhythmias (VAs)^7^ is still a matter of debate, and the true benefit of prophylactic ICD placement in women remains uncertain.^3^ Above all, none of these trials have analysed gender differences in S-ICD recipients, who constitutes a significantly different population in terms of clinical characteristics.

Women are often perceived as a more vulnerable group when considering invasive procedures, and the fear of device-related complications may contribute to limiting ICD implantation in female patients. Nevertheless, while transvenous ICDs carry the risk of significant lead-related complications especially in women,^8^ S-ICDs generally offer a lower rate and safer management of lead and major procedure-related complications, especially regarding lead extraction.^9^ However, to date, no data regarding sex differences in S-ICD technology have been reported. Therefore, the aim of this study was to evaluate sex-related differences regarding mid-term S-ICD outcomes in the largest independent, physician-initiated S-ICD registry, both in terms of appropriate therapy and device-related complications.

## Methods

The i-SUSI (International SUbcutaneouS Implantable cardioverter defibrillator registry)—former ELISIR project (NCT0473876)—is a multicentre, open-label, independent, and physician-initiated observational registry, whose composition has been previously described.^10^ Twenty-four public and private healthcare institutions from six different countries in Europe and in the USA are currently involved in the registry. All consecutive patients meeting current guideline indications for ICD implantation and undergoing implantation of an S-ICD device from 2011 to 2022 (Boston Scientific, Marlborough, MA, USA) were enrolled in the registry and used for the current analysis. This manuscript has been drafted in accordance with the tenets of the Helsinki Declaration and has been approved by the local institutional review board.

### Data collection, follow-up, and outcome definitions

Data collection methods for the patients enrolled in this registry have been previously presented.^11^ Briefly, from the overall registry population, all patients with a follow-up < 6 months from implantation were excluded, as well as patients with incomplete follow-up data. For each patient, the PRAETORIAN score was collected using either a two-projection post-procedural chest X-ray or intra-procedurally.^12,13^ Follow-up time zero was set at the time of S-ICD placement. Follow-up strategy was left to each centre’s policy, with most patients being evaluated at 1, 6, and 12 months, and every 6 months. An appropriate shock was defined as a therapy delivered because of a correctly recognized shockable rhythm. Inappropriate shocks were defined as shock delivered due to (1) a supraventricular tachycardia; (2) oversensing of either cardiac or non-cardiac signals; and (3) any other cause resulting in device shock in the absence of a clinical VA. Ineffective shocks were defined as failure to conversion to sinus after two shocks delivered to an appropriate shockable rhythm. As per registry protocol,^14^ other major device-related complications were defined according as follows: pocket haematoma requiring a transfusion or a pocket revision; pocket infection; lead displacement impacting device functioning and requiring reintervention; lead fracture; lead infection; device extraction; and unexpected pneumothorax.

### Study aims and cohort definition

**Figure 1:**
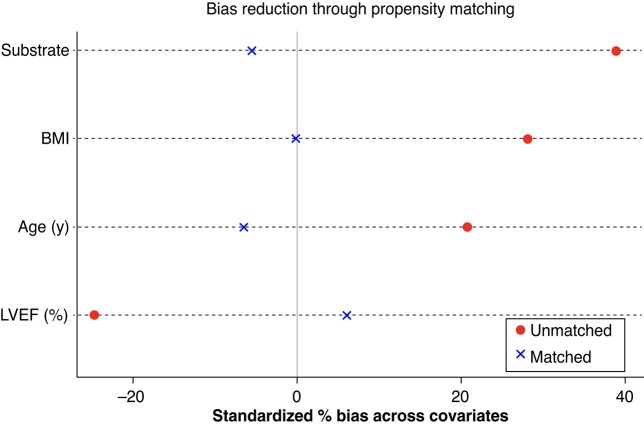
By covariate bias reduction with propensity matching.

Aim of the study was to report and compare the rates of appropriate shocks, inappropriate shocks, and device-related complications among S-ICD recipients, stratified by sex. For this purpose, two propensity-matched cohorts of males and females were retrieved. A 1:1 propensity matching was performed, addressing differences of clinical characteristics potentially affecting the study outcomes: age, body mass index (BMI), underlying substrate (stratified as previously reported^15^) and left ventricular ejection fraction (LVEF). *Figure 1* and *Table 1* report the pre- and post-propensity matching differences and the inter-cohort bias reduction. The primary outcome of the study was defined as comparing the rate of appropriate shocks observed throughout the entire follow-up period between the two sex groups. Additionally, rates of complications, inappropriate shocks, and cardiovascular mortality were assessed in both cohorts as secondary outcomes.

**Table 1:** Pre- and post-propensity matching differences and the inter-cohort bias reduction

Variable	Matched Unmatched	Value		% of bias reduction	*P* pre *P* post
		Male	Female		
Age (years)	U M	51.641 47.787	48.334 48.807	69.2%	<0.001 0.382
BMI (kg/m^2^)	U M	26.412 24.895	24.948 24.899	99.7%	<0.001 0.991
LVEF (%)	U M	41.825 46.618	45.785 45.634	75.2%	<0.001 0.416
Substrate	U M	0.365 0.176	0.194 0.201	85.9%	<0.001 0.401

BMI, body mass index; LVEF, left ventricular ejection fraction; M, matched; U, unmatched.

### Statistical analysis

Continuous variables were reported as mean ± standard deviation (SD) or as median [interquartile range (1st–3rd quartile) (IQR)] if normally or non-normally distributed, respectively. Categorical variables were reported as count (%). Propensity matching for the pre-specified variables was performed using the nearest neighbour method without replacement, using common support and a calibre set at 0.05. Comparisons have been performed using a χ^2^ test or a Fisher’s exact test between categorical variables, and a Student’s *t*-test or a Mann–Whitney *U* test between numerical variables, as appropriate according to their distribution. Event-free survival was plotted using Kaplan–Meier estimates and a log-rank test was used to compare them. A Cox regression was used to assess the associations between post-matching baseline and procedural characteristics and clinical outcomes. Time of censoring was set either as the time of the outcome or the time of last follow-up, whichever came first. Univariable analyses were performed at first, reporting unadjusted hazard ratios (HR); all variables reaching a threshold *P* value 0.10 were then fit into a multivariable model to adjust for confounders, from which adjusted hazard ratios (aHR) were retrieved. A two-sided *P* value < 0.05 was considered significant throughout the manuscript. All analysis was performed using STATA 14.0 (StataCorp LLC, College Station, TX).

## Results

### Overall i-SUSI population and substrate-specific cohorts

A total of 1698 patients were included in the i-SUSI registry, with 399 (23.5%) being female. After propensity matching, two cohorts of 374 matched patients each were enrolled for this study. Bias standardization through propensity matching has been shown in *Figure 1* and numerically reported in *Table 1*. Propensity matching normalized the differences in the underlying substrate, age, LVEF, and BMI.

*Table 2* reports baseline and implantation characteristics of the female and male S-ICD patient cohort. The baseline characteristics of the two post-matched cohorts were very similar, with a higher beta-blocker therapy at baseline in females (71.9% vs. 62.6%; *P* = 0.006) representing the only significant difference observed. Procedural characteristics were very similar between the two cohorts, with 92.8% women vs. 92.5% men (*P* = 0.889) and 81.6% vs. 84.5% (*P* = 0.284) undergoing an S-ICD placement with the two-incision technique in an intermuscular position, respectively. As for S-ICD programming characteristics, no significant differences were detected among the two cohorts, with the SMART-pass algorithm activated in *n* = 296 (79.1%) female and *n* = 300 male patients (80.2%), *P* = 0.716. The primary vector was programmed in *n* = 237 women (63.3%) vs. *n* = 242 (64.7%) men (*P* = 0.703), the secondary vector in *n* = 107 (28.6%) vs. *n* = 103 (27.5%) (*P* = 0.745), while the alternate vector in *n* = 30 (8.0) vs. *n* = 29 (7.8%) (*P* = 1.000). Interestingly, despite similar procedural characteristics and a matched BMI (females 24.9 ± 4.9 kg/m^2^ vs. males 24.9 ± 4.5 kg/m^2^, *P* = 0.991), the female S-ICD cohort had a lower rate of patients at a low-risk of failure conversion as per PRAETORIAN score (73.4% vs. 81.3%, *P* = 0.049). The overall need for a TV device upgrading in this propensity-matched cohort was 2.5%, with the main underlying reason being need for ventricular pacing (0.8%), while the need for ATP was only seen in 0.3% patients. No differences between sexes were found in this regard.

**Table 2:** Baseline and periprocedural characteristics of the study cohort

	Female (*n* = 374)	Male (*n* = 374)	*P*
Baseline characteristics			
Age (years), mean ± SD	48.8 ± 16.1	47.8 ± 15.8	0.382
BMI, mean ± SD	24.9 ± 4.9	24.9 ± 4.5	0.991
Diabetes, *n* (%)	46 (12.3)	40 (10.7)	0.492
Hypertension, *n* (%)	112 (29.9)	105 (28.1)	0.573
CKD, *n* (%)	53 (14.2)	40 (10.7)	0.150
LVEF (%), mean ± SD	45.6 ± 16.0	46.6 ± 17.0	0.416
Ischaemic cardiomyopathy, *n* (%)	75 (20.1)	66 (17.7)	0.400
Non-ischaemic cardiomyopathy, *n* (%)	299 (79.9)	308 (82.3)	
DCM, *n* (%)	78 (20.9)	87 (23.3)	0.427
HCM, *n* (%)	35 (9.4)	48 (12.8)	0.130
ARVC, *n* (%)	21 (5.6)	22 (5.9)	0.875
Primary electrical disease^a^, *n* (%)	107 (28.6)	110 (29.4)	0.809
Myocarditis, *n* (%)	19 (5.1)	15 (4.0)	0.483
Other, *n* (%)	39 (10.4)	26 (6.9)	0.089
Primary prevention implant, *n* (%)	217 (58.0)	217 (58.0)	1.000
Beta-blockers, *n* (%)	269 (71.9)	234 (62.6)	**0**.**006**
Procedural characteristics			
Numbers of suitable vectors, median [IQR]	2^2–3^	2^2–3^	1.000
Defibrillation test performed, *n* (%)	282 (76.8)	293 (79.2)	0.441
Two-incision technique, *n* (%)	347 (92.8)	346 (92.5)	0.889
Intermuscular placement, *n* (%)	305 (81.6)	316 (84.5)	0.284
Conditional zone, median [IQR]	200 [200–220]	200 [200–220]	0.284
Shock zone, median [IQR]	240 [230–250]	250 [240–250]	**0**.**032**
Standard shock polarity, *n* (%)	347 (92.8)	347 (92.8)	1.000
Vector used			
Primary, *n* (%)	237 (63.3)	242 (64.7)	0.703
Secondary, *n* (%)	107 (28.6)	103 (27.5)	0.744
Alternate, *n* (%)	30 (8.0)	29 (7.8)	0.891
PRAETORIAN score available, *n* (%)	244 (65.2)	240 (64.2)	0.760
PRAETORIAN score, median [IQR]	30 [30–60]	30 [30–60]	0.551
PRAETORIAN class			
Low, *n* (%)	180 (73.4)	195 (81.3)	**0.049**
Intermediate, *n* (%)	50 (20.5)	39 (16.3)	0.228
High, *n* (%)	14 (5.7)	6 (2.5)	0.073
SMART-pass algorithm on, *n* (%)	296 (79.1)	300 (80.2)	0.716
Remote monitoring, *n* (%)	318 (85.0)	318 (85.0)	1.000

ARVC, arrhythmogenic right ventricular cardiomyopathy; BMI, body mass index; CKD, chronic kidney disease; DCM, dilated cardiomyopathy; HCM, hypertrophic cardiomyopathy; IQR, interquartile range; LVEF, left ventricular ejection fraction; SVT, supraventricular tachycardia.

^a^Primary electrical disease = Brugada syndrome + long-QT syndrome + idiopathic ventricular fibrillation.

Bold indicates statistically significant values.

### Overall outcomes and sex differences

**Figure 2:**
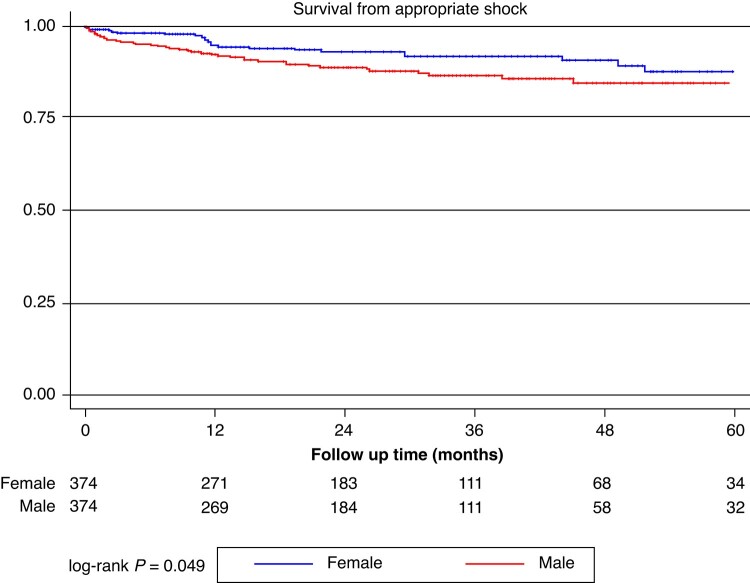
Kaplan–Meier curves showing overall freedom from appropriate device intervention in the female (blue) and male (red) cohort.

**Figure 3:**
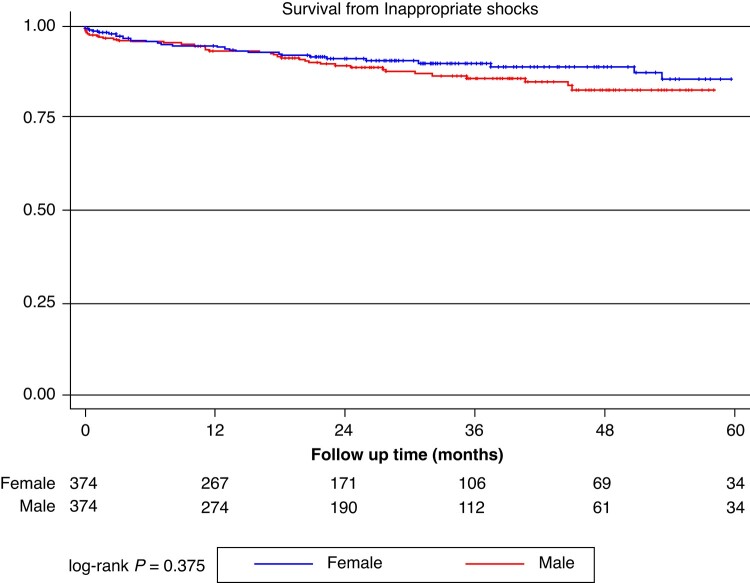
Kaplan–Meier curves showing overall freedom from inappropriate device intervention in the female (blue) and male (red) cohort.

**Figure 4:**
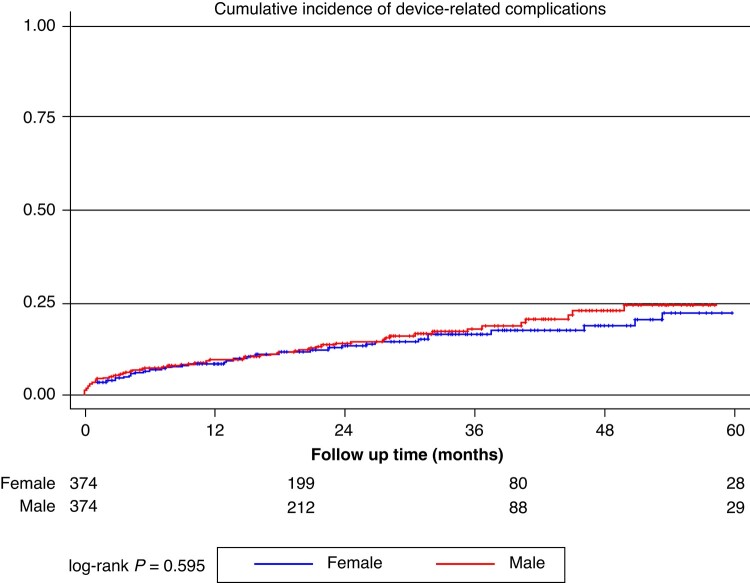
Kaplan–Meier curves showing overall freedom from device-related intervention in the female (blue) and male (red) cohort.

Over a median follow-up time of 26.5 [12.7–42.5] months (no differences between the cohorts: 25.3 [12.4–41.6] vs. 28.4 [13.8–42.9], *P* = 0.844), a total of 68 (9.1%) patients experienced appropriate shocks and 105 (14.0%) patients experienced a device-related complication, *n* = 75 (10.3%) of which had an inappropriate shock. *Figures 2–4* report Kaplan–Meier curves showing the rates of occurrence of those events in the male and female cohort. Appropriate shocks were more common in the male cohort (rate/year 3.4% vs. 1.7%; log-rank *P* = 0.049), while no significant differences in device-related complications (rate/year: 6.3% vs. 5.8%; log-rank *P* = 0.595) and inappropriate shocks (rate/year: 4.3% vs. 3.1% log-rank *P* = 0.375) were observed. *Table 3* details overall outcomes of both study cohorts.

**Table 3:** Clinical outcomes

Clinical outcomes	Female (*n* = 374)	Male (*n* = 374)	*P*
Follow-up time (months), median [IQR]	25.3 [12.4–41.6]	28.4 [13.8–42.9]	0.844
Appropriate shocks, *n* (%)	26 (7.0)	42 (11.2)	**0.042**
Ischaemic cardiomyopathy, *n* (%)	2 (0.5)	5 (1.3)	0.252
DCM, *n* (%)	4 (1.1)	8 (2.1)	0.379
HCM, *n* (%)	3 (0.8)	1 (0.3)	0.305
ARVC, *n* (%)	2 (0.5)	7 (1.9)	0.132
Primary electrical disease^a^, *n* (%)	8 (2.1)	15 (4.0)	0.138
Myocarditis, *n* (%)	1 (0.3)	2 (0.5)	0.571
Other, *n* (%)	6 (1.6)	4 (1.1)	0.524
Patients with device-related complications, *n* (%)	51 (13.6)	54 (15.6)	0.409
Inappropriate shocks, *n* (%)	32 (8.6)	43 (11.5)	0.181
SVT/AF, *n* (%)	6 (1.6)	6 (1.6)	1.000
T-waves oversensing, *n* (%)	11 (2.9)	21 (5.6)	0.071
Myopotentials, *n* (%)	6 (1.6)	7 (1.9)	0.780
Other, *n* (%)	9 (2.4)	9 (2.4)	1.000
Lead displacement, *n* (%)	5 (1.3)	1 (0.3)	0.217
Lead rupture, *n* (%)	1 (0.3)	1 (0.3)	1.000
Lead infection, *n* (%)	2 (0.5)	4 (1.1)	0.686
Pocket infection, *n* (%)	4 (1.1)	4 (1.1)	1.000
Pocket haematoma, *n* (%)	10 (2.7)	10 (2.7)	1.000
Ineffective shocks, *n* (%)	5 (1.3)	3 (0.8)	0.725

AF, atrial fibrillation; ARVC, arrhythmogenic right ventricular cardiomyopathy; DCM, dilated cardiomyopathy; HCM, hypertrophic cardiomyopathy; SVT, supraventricular tachycardia.

^a^Primary electrical disease = Brugada syndrome + long-QT syndrome + idiopathic ventricular fibrillation.

Bold indicates statistically significant values.

### Predictors of outcomes

When considering the primary outcome of our analysis, male sex was associated with increased rates of appropriate shocks at Cox regression analysis (aHR 1.648; CI [0.999–2.655]; *P* = 0.048), while primary prevention implantation (aHR 0.450; CI [0.271–0.741], *P* = 0.002) was associated with lower rates of appropriate shocks. Sex differences were not predictors of either device-related complications or inappropriate shocks; instead, predictors included age (aHR for inappropriate shocks 0.972; CI [0.955–0.989]; *P* = 0.001, aHR for overall device-related complications 0.980; CI [0.964–0.995]; *P* = 0.009) and the use of the SMART-pass algorithm (aHR for inappropriate shocks 0.53; CI [0.299–0.948]; *P* = 0.032). BMI resulted another predictor of overall device-related complications (aHR 1.071; CI [1.024–1.119]; *P* = 0.002). *Table 4* reports the univariate and multivariate assessment of the predictors of primary and secondary outcomes of our study.

**Table 4:** Study outcome predictors

Appropriate shock predictors						
Age	0.979	[0.964–0.994]	0.006	0.986	[0.970–1.002]	0.096
Male sex	1.664	[1.015–2.699]	0.040	1.648	[0.999–2.655]	0.048
Hypertension	0.689	[0.393–1.234]	0.220			
BMI	1.012	[0.962–1.065]	0.634			
Diabetes	0.859	[0.369–2.003]	0.726			
CKD	0.676	[0.292–1.564]	0.360			
LVEF	1.003	[0.989–1.018]	0.647			
Primary prevention	0.404	[0.245–0.665]	<0.001	0.450	[0.271–0.741]	0.002
Shock zone	0.994	[0.971–1.014]	0.591			
Ischaemic substrate	0.484	[0.221–1.059]	0.069	0.668	[0–295–1.515]	0.334

Device-related complication predictors						
Age	0.984	[0.971–0.999]	0.034	0.980	[0.964–0.995]	0.009
Male sex	1.300	[0.811–2.084]	0.276			
Hypertension	1.109	[0.654–1.881]	0.700			
BMI	1.055	[1.008–1.104]	0.020	1.071	[1.024–1.119]	0.002
Diabetes	0.994	[0.470–2.100]]	0.987			
CKD	1.156	[0.567–2.357]	0.690			
LVEF	0.997	[0.984–1.011]	0.666			
Shock zone	1.007	[0.984–1.032]	0.529			
Ischaemic substrate	0.779	[0.447–1.357]	0.379			

Inappropriate shock predictors						
Age	0.972	[0.955–0.989]	0.002	0.972	[0.955–0.989]	0.001
Male sex	1.506	[0.847–2.675]	0.162			
Hypertension	0.783	[0.403–1.520]	0.470			
BMI	1.029	[0.973–1.088]	0.311			
Diabetes	0.912	[0.356–2.331]	0.847			
CKD	0.480	[0.148–1.558]	0.222			
LVEF	1.001	[0.984–1.016]	0.981			
Shock zone	1.017	[0.986–1.049]	0.283			
SMART-pass algorithm on	0.543	[0.304–0.967]	0.038	0.531	[0.299–0.948]	0.032
Ischaemic substrate	0.665	[0.333–1.328]	0.248			

BMI, body mass index; CKD, chronic kidney disease; LVEF, left ventricular ejection fraction.

## Discussion

The aim of this study was to analyse sex-related differences among S-ICD recipients enrolled in a large, multicentre, international registry. The main results of our analysis are hereby summarized:

- In a large, multicentre, real-world registry encompassing a broad population of 1698 patients, women represented the 23.5% of the entire cohort.
- After 1:1 propensity matching for age, BMI, LVEF, and underlying arrhythmic substrate, appropriate shocks were more common in the male cohort. Male sex remained an independent predictor of appropriate device therapy even after controlling for confounders, while the overall rate of device-related complications and the rate of inappropriate shocks were comparable between sexes.
- Despite similar procedural characteristics and a matched BMI, female S-ICD carriers were more likely to be at low-risk of VAs conversion failure as per PRAETORIAN score.

### Women and subcutaneous implantable cardioverter-defibrillator: underrepresented or undertreated?

In our observational study, enrolling unselected consecutive S-ICD patients, women represented the 23.5% of the entire cohort. This overall low rate of women enrolled is in line with other S-ICD trials, like the UNTOUCHED^16^ (25.6% of females enrolled) and the PRAETORIAN trial^17^ (19.6%), as well as with other S-ICD post-approval studies (24–32%).^2,18^ This relatively low incidence of women receiving S-ICDs may suggest a tendency towards favouring a less invasive approach when treating/preventing life-threatening VAs in the female cohort, especially considering that receiving the S-ICD in primary prevention represents its most common indication. Divergent findings have been documented regarding the influence of gender on the susceptibility to VAs and the necessity for ICD therapies. While certain studies have indicated non-significant distinctions,^19^ others have demonstrated gender-specific risk disparities,^20^ with women exhibiting up to a 35% lower risk of VAs and a 41% lower risk of appropriate ICD therapies.^21,22^

Since differences in clinical characteristics between females and males may have led to biased conclusions, even in extensive observational investigations, we appropriately used propensity matching to account for the underlying substrate and LVEF differences. Despite adjusting for clinical baseline differences that may have accounted for differences in appropriate shocks, we found that also in S-ICD carriers there is a disparity regarding the VA rate, conditioning more frequent appropriate device interventions in the male cohort (rate/year 3.4% vs. 1.7%; log-rank *P* = 0.049). However, it should be noted that despite propensity matching mitigating significant differences among groups, there was still higher beta-blocker usage in the female cohort, although no baseline differences were noted in the rate of HF patients enrolled for this analysis. This might, at least partially, have influenced the primary study outcome, potentially accounting for the higher appropriate shock rate in the female cohort. Underlying electrophysiological properties, anatomical differences (i.e. the smaller volume of the heart chambers), as well as hormonal regulation may have an impact on men’s greater arrhythmic vulnerability.^23^ Saxena *et al*.^3^ demonstrated that women implanted with a TV-ICD had this lower risk of VAs (when compared to their male counterpart), especially pronounced in patients with non-ischaemic cardiomyopathies compared with those with ischaemic cardiomyopathies. Since the S-ICD device is frequently implanted in non-ischaemic patients,^2,15^ this may have influenced our results, despite accounting for baseline differences. However, why women represented only a marked minority of device recipients in S-ICD cohorts is still unclear. We believe that the exhibited lower VA risk may have played a significant role, even though unrevealed sex disparities in implantation rates in ordinary practice for other reasons might not be excluded. Identifying females at high risk for malignant VAs prior to device implantation remains challenging, yet of significant importance.

### Sex differences and device-related complications

Although no reports specifically assessed sex differences among S-ICD carriers, the impact of sex regarding device-related complications has been briefly analysed in two previous S-ICD studies. Gold *et al*.^18^ reported that women were more likely to have device-related complications (15.7% of female patients vs. 10.4% of male patients, *P* < 0.005), including haematoma, pulse generator movement, and inadequate healing of the incision site. Boersma *et al*.^24^ reported only more frequent device repositioning rate secondary to electrode migration in females, but this finding did not reach statistical significance. In our analysis, we did not find any difference neither in terms of device-related complications (rate/year: 6.3% vs. 5.8%; log-rank *P* = 0.595) nor in terms of inappropriate shocks (rate/year: 4.3% vs. 3.1% log-rank *P* = 0.375) between cohorts. The most likely explanation for this finding is that we accounted for baseline BMI differences with propensity matching, suggesting that more than an effect of sex *per se*, a different body habitus may eventually represent the major determinant of device-related complications. In accordance to what reported in the S-ICD post-approval studies,^18,25^ our previous analysis clearly show that a higher BMI is also strongly associated with a higher complication rate, impacting both infective and non-infective ones, as already reported.^14^ This suggests that the distribution of subcutaneous fat tissue may contribute to a higher number of complications during follow-up, indicating that factors driving complications should be investigated beyond gender. Certainly, crafting a suitable pocket in individuals with a greater BMI could pose a challenge, potentially increasing the risk of pocket haematomas and/or infections, irrespective of gender. Implant techniques have advanced to the point where the necessary surgical skills are well-acknowledged and the intermuscular technique becomes the standard of care (81.6% and 84.5% in females and males, respectively). This approach potentially reduces electrode migration and pocket discomfort in females, who typically have less subcutaneous tissue.

Previous studies on TV devices have clearly shown an increased likelihood of complications such as pneumothorax, pocket haematomas, and lead perforation in women undergoing TV-ICD implantation,^8,26^ despite it being traditionally considered a less complex and faster procedure than S-ICD implantation. Apart for their smaller body size, women may exhibit anatomical features that pose greater technical challenges, such as a thinner right ventricular wall and smaller blood vessel diameter, which may predispose them to adverse events during and after TV-device implantation. In the light of our results, the S-ICD should be deemed a valid alternative in such cases, whenever appropriate. Future enhancements could arise from refined patient selection, technological advancements (e.g. smaller device cans), increased operator expertise, and optimization of perioperative care.

Furthermore, no sex differences regarding infective events during follow-up have been noted during TV-ICD patients follow-up (2.2% males vs. 1.8% females over a large Medicare database^27^). The infection rate in our cohort (2.6% females vs. 2.1% males) is similar to that noted in other S-ICD^18^ and TV-ICD studies,^27^ over similar follow-up duration. However, as expected, there were no instances of bacteraemia arising from the S-ICD infection over the follow-up period.

Lastly, no differences between sexes were found in terms of needing an upgrading to a transvenous device, in line to what reported in a focused analysis from the i-SUSI registry on this topic.^10^ Thus, sex was neither a predictor of the overall need for a TV-device upgrade (OR 0.790 CI [0.400–1.580], *P* = 0.505) nor of the need for TV upgrade due to pacing requirements (OR 1.070, CI [0.410–2.770], *P* = 0.896) in the overall cohort.

### Sex differences and PRAETORIAN score

Despite a matched BMI and no differences regarding periprocedural characterizes, females resulted at lower risk of conversion failure as per PRAETORIAN score when compared to their male counterpart (73.4% vs. 81.3%, *P* = 0.049). This is the first study reporting this relevant clinical finding, since PRAETORIAN score validation studies^28^ and sub-analysis^29^ did not address specific sex differences regarding the score. Since there were no differences in BMI among groups after propensity matching, this may indicate that the operators were able to achieve less interposition of fat tissue between the nearest point of the generator and the thoracic wall, as well as a more posterior positioning of the generator in females. This finding, along with the equal number of complications when compared to their male counterparts, unlike what occurs with TV-ICDs, highlights the urgency of bridging the gap of underrepresentation of women referred for S-ICD implantation.

Whether this greater representation in the low-risk group according to PRAETORIAN score stratification may translate into a lower risk of ineffective shocks is yet to be determined. In our cohort, we did not find any significant differences (1.3% vs. 0.8%, *P* = 0.725) in this regard, even though due to the overall number of events our study might be underpowered to detect differences for this clinical outcome. However, our findings are in line with what was reported by Doldi *et al*.^29^ in terms of the absence of sex differences as a predictor of ineffective shocks.

### Limitations

The primary limitation of our study lies in its non-randomized, observational format, which is an inherent aspect of the real-world, multicentre registry involving unselected patients who underwent S-ICD implantation, from which our data were derived. However, the strength of our study lies in its large-scale real-world nature, which likely mitigated some degree of selection bias, providing accurate data on the actual clinical practice scenario; moreover, the use of propensity matching may have contributed to hamper selection bias as well. Secondly, all institutions involved in this study are highly specialized electrophysiology centres for S-ICD implantation, where all procedures are conducted by experienced electrophysiologists. Therefore, our clinical outcomes may not be representative of the results obtained by less-experienced operators, especially regarding PRAETORIAN scores and periprocedural complications. Thirdly, due the relatively low number of ineffective shocks, this analysis might be, at least partially, underpowered to detect sex differences regarding this clinical outcome.

## Conclusion

In a large, multicentre, real-world registry of S-ICD recipients, women were significantly underrepresented. After propensity matching accounting for baseline differences, women were less likely to experience appropriate ICD therapy, while not showing higher risk of device-related complications. Female S-ICD carriers were more likely to be at a low-risk of VAs conversion failure as per PRAETORIAN score.

## Supplementary Material

euae115_Supplementary_Data

## Data Availability

The data that support the findings of this study are available from the corresponding author upon reasonable request.
