# Supplementary material for: Sex differences among subcutaneous implantable cardioverter-defibrillator recipients: a propensity-matched, multicentre, international analysis from the i-SUSI project
Source: Europace. 2024 May 2;26(5):euae115. doi: 10.1093/europace/euae115 (PMC11100525; doi:10.1093/europace/euae115)
Supplement: euae115_Supplementary_Data [file euae115_supplementary_data.docx]

**Appendix - List of participating centers and investigators:**

**Italy**

Ospedale Luigi Sacco (Polo Universitario) – Milan: A. Gasperetti, R. Arosio, M. Viecca, G.B. Forleo

Centro Cardiologico Monzino – Milan: M. Schiavone, F. Tundo, M. Moltrasio, C. Tondo

Policlinico Sant’Orsola Malpighi – Bologna: M. Ziacchi, I. Diemberger, A. Angeletti, M. Biffi

Ospedale San Raffaele – Milan: N. Fierro, S. Gulletta, P. Della Bella

Spedali Civili Brescia – Brescia: G. Mitacchione. A. Curnis

Ospedale “Umberto I-Salesi-Lancisi” – Ancona: P. Compagnucci, M. Casella, A. Dello Russo

Ospedale G.B. Grassi – Ostia: L. Santini

Ospedale San Filippo Neri – Rome: C. Pignalberi

Policlinico Umberto I – Rome: M. Magnocavallo, A. Piro, C. Lavalle

Campus Biomedico – Rome: F. Picarelli, D. Ricciardi

Policlinico Casilino – Rome: E. Bressi, L. Calò

Ospedale San Gerardo – Monza: E. Montemerlo, G. Rovaris

Ospedale di Castrovillari – Castrovillari: S. De Bonis, A. Bisignani, G. Bisignani

Ospedale Vito Fazzi – Lecce: G. Russo, E. Pisanò

Ospedale Card. Panico – Tricase: P. Palmisano

Ospedale Santa Chiara – Trento: F. Guarracini

AOU Ferrara - Arcispedale S.Anna – Ferrara: F. Vitali, M. Bertini

**Germany**

UKSH Universitätsklinikum Schleswig-Holstein – Lübeck: J. Vogler, T. Fink, R. Tilz

Universitätsklinik Mannheim – Mannheim: F. Fastenrath, J. Kuschyk

Asklepios Klinik St. Georg – Hamburg: L. Kaiser, S. Hakmi

**France**

APHP, Hôpital Pitié Salpêtrière – Paris: M. Laredo, X Waintraub, E. Gandjbakhch, N. Badenco

**Switzerland**

Universitätsspital Zürich – Zurich: A. Breitenstein, A.M. Saguner

**Austria**

Ordensklinikum Linz Elisabethinen – Linz: M. Martine, S. Seidl

**USA**

Montefiore Einstein Center for Heart and Vascular Care, Montefiore Medical Center, Albert Einstein College of Medicine – New York: X. Zhang, L. Di Biase
